# Gestational Melatonin Supplementation Attenuates Maternal Sleep Deprivation‐Induced Steatohepatitis Susceptibility in Offspring

**DOI:** 10.1111/cpr.70138

**Published:** 2025-10-28

**Authors:** Fei Guo, Zexin Yang, Junsen She, Chen Fang, Yizhi Hu, Hefeng Huang, Ling Gao

**Affiliations:** ^1^ Obstetrics & Gynecology Hospital of Fudan University, Shanghai Key Lab of Reproduction and Development, Shanghai Key Lab of Female Reproductive Endocrine Related Diseases, Institute of Reproduction and Development, Fudan University Shanghai China; ^2^ International Institutes of Medicine, the Fourth Affiliated Hospital, Zhejiang University School of Medicine Yiwu China; ^3^ Institute of Medical Genetics and Development, Key Laboratory of Reproductive Genetics (Ministry of Education) and Women's Hospital, Zhejiang University School of Medicine Zhejiang China; ^4^ Research Units of Embryo Original Diseases, Chinese Academy of Medical Sciences (No. 2019RU056) Shanghai China

**Keywords:** inflammation, melatonin, nonalcoholic steatohepatitis, offspring, sleep deprivation

## Abstract

Sleep deprivation (SD) is a common issue among pregnant women. Maternal SD led to adverse effects on offspring health such as cognitive impairment through dysregulated metabolic pathways. However, it remains unknown whether maternal SD increases the offspring's susceptibility to nonalcoholic steatohepatitis (NASH) development. Here, we induced maternal SD during pregnancy and observed that maternal SD during pregnancy promoted the development of diet‐induced NASH in offspring of both sexes in adulthood, with exacerbation of liver weight gain, hepatic steatosis, fibrosis, and hepatic dysfunction. The primary hepatocytes isolated from SD offspring were also more susceptible to palmitate acid‐induced lipotoxic injury. Mechanistically, the detrimental effects of maternal SD were associated with augmented activation of inflammatory and apoptosis pathways in offspring liver tissues, which were attributed to upregulation of the transcription factor nuclear receptor subfamily 4 group A member 3 (NR4A3). The melatonin signalling is reported to be pivotally affected by sleep disturbance both at the circulation and the placenta, and our further analysis revealed that melatonin supplementation during maternal SD normalised NR4A3 expression in offspring liver and alleviated the increased steatohepatitis susceptibility in offspring. Taken together, these results suggest that maternal SD during pregnancy predisposes offspring to NASH development in adulthood via an NR4A3‐dependent mechanism, and maternal melatonin supplementation may hold promise for improving liver health in the offspring.

## Graphical abstract

Maternal SD during pregnancy enhances the development of diet‐induced NASH in adult offspring of both sexes through an NR4A3‐dependent mechanism, characterised by heightened inflammation and increased apoptosis. Maternal melatonin supplementation effectively alleviates this increased susceptibility to NASH in offspring.

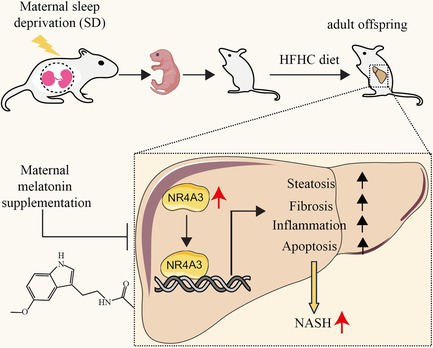

## 1 Introduction

Non‐alcoholic fatty liver disease (NAFLD) is the most common chronic liver disease worldwide [1]. It encompasses a spectrum of liver disorders, including simple steatosis, nonalcoholic steatohepatitis (NASH), and cirrhosis [2]. The prevalence of NAFLD is increasing and afflicting over 30% of the global population [3, 4]. In particular, NASH is an advanced NAFLD form characterised by the presence of hepatic steatosis, hepatocellular damage, signs of inflammation and fibrosis [5]. Notably, NASH increases the risk of type 2 diabetes mellitus, cardiovascular diseases, hypertension and chronic kidney disease [6, 7]. However, effective treatment for NASH remains largely unmet [8]. Emerging epidemiological studies and animal models have revealed that adverse prenatal environments have significant long‐term influences on offspring health [9], including the development of NASH [10, 11]. The offspring from obese mothers had significantly increased lipid accumulation in the liver [12], and the inflammation markers were markedly upregulated in the liver from the offspring of high fat diet‐fed fathers [13], indicating NASH has an early‐life origin. Therefore, investigating the pathogenesis of NASH from a developmental perspective may provide new insights into the management of NASH.

Sleep deprivation (SD) is highly prevalent, with approximately 77% of pregnant women reporting sleep disturbances [14], and many reporting suffering from insomnia in the third trimester of pregnancy [15]. The implications of SD during pregnancy extend beyond maternal discomfort, inducing neurogenesis inhibition [16], cognitive impairment [17], hypertension [18] and hyposexuality [19] in the offspring. However, there is no direct evidence on the effects of maternal SD during pregnancy on NASH susceptibility in their offspring.

Melatonin is an endogenous circadian hormone synthesised in the pineal gland, recognised for its roles in maintaining circadian rhythms [20], and it also possesses powerful anti‐inflammatory and antioxidant properties [21]. Melatonin secretion is affected by sleep disturbance [22], and the melatonin signalling in the placenta is inhibited by SD during pregnancy [23]. Of note, melatonin supplementation during SD in pregnant rodents improved cognitive impairment and downregulated brain inflammation in adult offspring [24]. Therefore, the current study aimed to investigate whether the offspring from maternal SD during pregnancy are more susceptible to NASH in adulthood, and whether gestational melatonin treatment exerts a beneficial effect.

## 2 Materials and Methods

### 2.1 Animals

All the animal experiments were approved by the Institutional Animal Ethics Committee and were performed according to the National Institutes of Health Guidelines on the Use of Laboratory Animals. C57BL/6J female mice were adapted for 1 week in the animal facility, and then mated with male mice. Pregnant mice were subjected to SD or control treatment. Male and female offspring were included for further studies and were fed a normal control diet (NC, Shuyu Biotechnology) or a high fat, high fructose, high cholesterol diet (HFHC, Shuyu Biotechnology, Cat# SYHFHFrC01) consisting of 20% protein, 40% fat, and 40% carbohydrates and containing 2% cholesterol for 16 weeks. All animals were housed under 12‐h light/12‐h dark, at a temperature of 24°C ± 2°C and humidity of 40% ± 5%, and with freely available food and water.

### 2.2 Sleep Deprivation

We used the multi‐platform water environment method to induce the SD model [25]. The SD experiment began from gestational day 14 (GD14) to parturition. For the SD group, platforms of 3 cm in diameter were evenly placed inside the cage. If the mice entered to sleep, they would fall into the water from the platforms and wake up. For the control group, the cages were equipped with wide platforms of 12 cm in diameter. The daily SD period was 16 h (from 6:30 AM to 10:30 PM), and afterwards, the SD mice were allowed to rest. To observe the effects of melatonin supplementation, melatonin was administered by intraperitoneal injection daily from GD14 to parturition at 10 mg/kg of body weight. The control mothers were injected intraperitoneally with vehicle.

### 2.3 Energy Expenditure Assessment

The offspring from both groups were individually housed in a comprehensive lab animal monitoring system (CLAMS) metabolic cage 1 day prior to data collection for 24‐h acclimation. Following adaptation, the mice were continuously monitored for 72 h under constant conditions. Parameters including gas exchange, energy consumption, daily food, and water intake were recorded. Throughout the experiment, all the mice were free to access food and water.

### 2.4 Glucose Tolerance Test (GTT)

For GTT, the mice were fasted for 12 h with free access to water. Afterwards, the mice received an intraperitoneal injection of glucose (2 g/kg body weight). Blood samples were collected and tested at baseline, 30, 60, 90, and 120 min post‐injection, respectively.

### 2.5 Body Mass Analysis by Dual‐Energy X‐Ray Absorptiometry

The iNSiGHT VET dual‐energy X‐ray absorptiometry (DXA, Osteosys, Korea) was used to measure the body fat. Mice were anaesthetised by isoflurane and placed on a plate in a prostrate position for the scan. The embedded algorithm was used to calculate the body composition.

### 2.6 Histological Analysis

Mice were sacrificed under adequate anaesthesia, and the livers were excised, weighed, fixed in 4% formalin, and embedded in paraffin or optimum cutting temperature (OCT) freeze medium. The samples were cut into 5‐um sections and then stained with haematoxylin and eosin (HE), oil‐red O and Sirius red staining for assessment of steatosis and fibrosis. Images were captured using a light microscope (Leica, Germany). Steatosis and inflammation scores were calculated based on the histological results using a recognised scoring system for NAFLD models [26].

### 2.7 Biochemical Analysis

The levels of alanine aminotransferase (ALT) and aspartate amino transferase (AST) in the serum were measured with an automated biochemical analyser (Rayto Chemray 240, China). Triglyceride (TG) concentration in the liver was measured by the commercially available kit (Applygen Technologies Inc., Cat# E1013).

### 2.8 Measurement of Apoptosis

Hepatic apoptosis was measured with a terminal deoxynucleotidyl transferase dUTP nick‐end labeling (TUNEL) staining kit (Roche Diagnostics, REF# 11684795910). After fixation and permeabilisation, the cells or slices were incubated in TUNEL reaction mixture for 1 h, then the nuclei were stained by DAPI. The apoptotic index was calculated as the percentage of TUNEL‐positive hepatocyte numbers over the total hepatocyte number.

### 2.9 Hydroxyproline Measurement

The hepatic hydroxyproline contents were measured by the Hydroxyproline Assay Kit according to the manufacturer's instructions (Elabscience Biotechnology, Cat# E‐BC‐K062‐M). Briefly, the frozen liver tissues (100 mg) were cut into small cubes and acid‐hydrolysed with HCl (6 mol/L) at 95°C for 6 h. Then, the working solution was added, and the supernatant was used for the colorimetric assay.

### 2.10 Flow Cytometry Analysis

Mice were sacrificed and perfused with 50‐mL PBS to remove the residual blood. Livers were weighed, cut into small cubes, and digested with 1 mg/mL collagenase IV for 30 min at 37°C. The cells were collected and centrifuged, then red blood cells were lysed. The live/dead cells were stained with Zombie UV Fixable Viability Kit (Biolegend, Cat# 423107). The following antibodies were used for flow cytometric analysis: anti‐mouse CD45‐PE/Cyanine7, anti‐mouse CD11b‐FITC, anti‐mouse F4/80‐PE, anti‐mouse F4/80‐BV421, anti‐mouse Ly‐6G‐Brilliant Violet 421, anti‐mouse Ly‐6C‐PerCP/Cyanine5.5, anti‐mouse GR‐1‐PE, anti‐mouse CD3‐PE, anti‐mouse CD19‐PE, anti‐mouse NK1.1‐BV421, anti‐mouse CD49b‐FITC. Absolute cell counts were calculated by adding Precision Count Beads (Biolegend, Cat # 424902) in samples.

### 2.11 Primary Hepatocyte Culture and Treatment

After euthanasia of the mice, a catheter was inserted into the inferior vena cava and perfused with phosphate buffered saline (PBS), then perfused with collagenase IV digestion solution (0.5 mg/mL, Worthington, Cat# LS004188). After adequate digestion, the liver was removed, torn apart, and digested in the medium. The suspension was passed through a 70‐μm cell strainer and centrifuged at 50 g for 2 min. Then the hepatocytes were resuspended in DMEM medium supplemented with 10% fetal bovine serum and cultured at 37°C containing 5% CO2 in the air. Lipotoxicity was induced by palmitic acid treatment (PA, Sigma‐Aldrich, Cat# P0500) at 0.5 mM for 24 h. To overexpress or silence NR4A3 in primary hepatocytes, the hepatocytes were transfected with adenovirus vectors encoding *Nr4a3* or relevant shRNA (Hanbio, Shanghai, China).

### 2.12 Assays in Primary Hepatocytes

Lactate dehydrogenase (LDH) release was detected by CytoTox 96 Non‐Radioactive Cytotoxicity Assay (Promega, Cat# G1780). Briefly, 50‐μL hepatocyte culture supernatant was transferred into a 96‐well plate, followed by sequential addition of working solutions. Finally, the absorbance value was measured at 490 nm in a plate reader. Mitochondrial membrane potential was detected using a 5,5′,6,6′‐tetrachloro‐1,1′,3,3′‐tetraethyl‐imidacarbocyanine iodide (JC‐1) staining method (Beyotime, Cat# C2003S). The cellular lipid accumulation was measured by Oil Red O staining (Sangon, Cat# A600395).

### 2.13 RNA‐Seq Library Preparation and Sequencing

The total RNA of liver tissues from male offspring was extracted according to standard instructions, and RNA integrity assessment was performed by Agilent 2100. The libraries were constructed and sequenced on the Illumina HiSeq X Ten platform. Clean reads were aligned against the mouse genome (GRCm38) using hisat2, and the read counts per gene were obtained by htseq‐count. The DESeq package was used to identify the gene expression changes, and differentially expressed genes (DEGs) were defined as the genes with a fold change > 1 and adjusted *p* values < 0.05. Gene Ontology (GO) enrichment analysis of DEGs was conducted in R using the clusterProfiler library [27]. Gene set enrichment analysis (GSEA) was performed, and the normalised enrichment score for each gene set was calculated.

### 2.14 Reverse Transcription‐Polymerase Chain Reaction (RT‐PCR)

Total RNA was extracted from the liver tissue and hepatocyte using TRIzol (Takara, Cat# 9108). Following the synthesis of cDNA, RT‐PCR was performed after adequate mixture of cDNA, forward and reverse primers, SYBR Green mixture, and RNase‐free water. The relative quantification of mRNA levels was calculated by the 2^−ΔΔCt^ method. The primer sequences used were as follows: mouse Nr4a3, forward 5′‐AGGGCTTCTTCAAGAGAACGG‐3′ and reverse 5′‐CCATCCCGACACTGAGACAC‐3′; mouse Il1β, forward 5′‐TCGCAGCAGCACATCAACAAG‐3′ and reverse 5′‐TCCACGGGAAAGACACAGGTAG‐3′; mouse Il2, forward 5′‐GGACCTCTGCGGCATGTTCTG‐3′ and reverse 5′‐TCCACCACAGTTGCTGACTCATC‐3′; mouse Il6, forward 5′‐GAGAGGAGACTTCACAGAGGATACC‐3′ and reverse 5′‐TCATTTCCACGATTTCCCAGAGAAC‐3′; mouse Tnfα, forward 5′‐CACGCTCTTCTGTCTACTGAACTTC‐3′ and reverse 5′‐CTTGGTGGTTTGTGAGTGTGAGG‐3′; mouse Cxcl1, forward 5′‐GCTGGGATTCACCTCAAGAACATC‐3′ and reverse 5′‐GTGTGGCTATGACTTCGGTTTGG‐3′; mouse Cxcl2, forward 5′‐GACAGAAGTCATAGCCACTCTCAAG‐3′ and reverse 5′‐TCAGTTAGCCTTGCCTTTGTTCAG‐3′; mouse Ccl2, forward 5′‐CACTCACCTGCTGCTACTCATTC‐3′ and reverse 5′‐GCTTCTTTGGGACACCTGCTG‐3′; mouse Ccl3, forward 5′‐CTCCCAGCCAGGTGTCATTTTC‐3′ and reverse 5′‐GGCATTCAGTTCCAGGTCAGTG‐3′; mouse Ccl4, forward 5′‐CTGCGTGTCTGCCCTCTCTC‐3′ and reverse 5′‐GCAGGAAGTGGGAGGGTCAG‐3′; mouse Ndufs3, forward 5′‐GATGTCCCAACTCGGCAGAA‐3′ and reverse 5′‐GTCCCAGACCTCCCTCTCAT‐3′; mouse Uqcrfs1, forward 5′‐ATGCGGCCAAAAATGTGGTC‐3′ and reverse 5′‐TATGGCGCACAAACAGAGGT‐3′; mouse Cox5a, forward 5′‐GATGCTCGCTGGGTGACATA‐3′ and reverse 5′‐AACCGTCTACATGCTCGCAA‐3′; mouse Cox5b, forward 5′‐TCTAGTCCCGTCCATCAGCA‐3′ and reverse 5′‐CCTTTGTGCAGCCAAAACCA‐3′; β‐actin, forward 5′‐TGAGAGGGAAATCGTGCGTGAC‐3′ and reverse 5′‐GGAAGAGGATGCGGCAGTGG‐3′.

### 2.15 Western Blot

Proteins were extracted by lysis, followed by centrifugation of the lysates at 12000 g for 5 min at 4°C to obtain the supernatant. For quantification of cytochrome c release, cytosolic and mitochondrial fractions were separated as previously described [28]. The BCA protein assay kit (Beyotime, Cat# P0012) was used to determine protein concentration and the equal amounts of total protein were loaded to sodium dodecyl sulphate–polyacrylamide gel electrophoresis (SDS‐PAGE). After electrophoresis, proteins were transferred onto PVDF membranes and then blocked with 5% skim milk at room temperature for 1 h. Subsequently, the membrane was incubated with specific primary antibodies overnight at 4°C: NR4A3 (1:1000; Abcam, Cat# ab94507), cleaved CASPASE3 (1:1000; Affinity, Cat# AF7022), BAX (1:1000; Affinity, Cat# AF0120), BCL‐2 (1:1000; Affinity, Cat# AF6139), Cytochrome C (1:1000; CST, Cat# 12959), GAPDH (1:10000; Affinity, Cat# AF7021) and beta‐Actin (1:1000; Abcam, Cat# ab8224). After incubation with the corresponding secondary antibodies, the protein bands were visualised using enhanced chemiluminescence. The bands were quantified with Image J software.

### 2.16 Statistical Analysis

The normality of continuous variables was tested by the Q‐Q plot and Shapiro–Wilk test, and the homogeneity of variance was tested by Levene's test. The differences between two groups were analysed by Student's *t*‐test and the Mann–Whitney *U* test. One‐way analysis of variance (ANOVA) or two‐way ANOVA was employed to compare the differences among multiple groups. All statistical analyses were performed using R version 3.6.0. *p* < 0.05 was considered significant.

## 3 Results

### 3.1 Maternal Sleep Deprivation During Pregnancy Promoted the Development of NASH in Offspring

**FIGURE 1:**
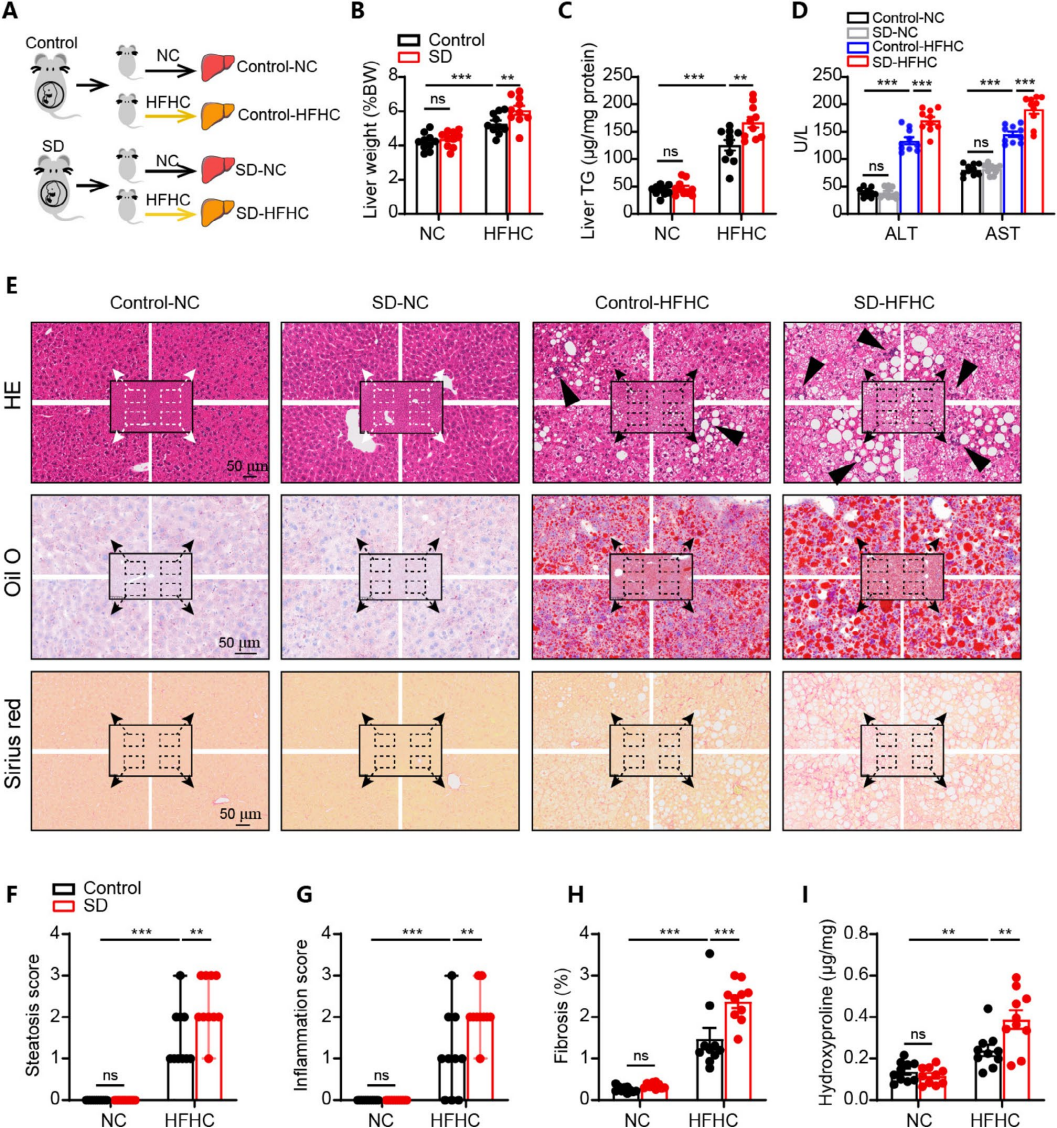
Maternal sleep deprivation exacerbated the progression of non‐alcoholic steatohepatitis (NASH) in male offspring. (A) Schematic illustration showing the experimental protocol. (B–D) Comparison of liver weight/body weight ratios, liver TG contents, and serum levels of ALT and AST in the offspring from different groups. (E) Representative images of the liver sections stained with haematoxylin–eosin (HE), oil red O and Sirius red in indicated groups. Scale bar represents 50 um. Black arrow heads indicate infiltrated immune cells. (F, G) Steatosis scores and inflammation scores based on liver histological staining in the offspring from different groups. (H) Fibrotic areas in the liver based on Sirius red staining in the offspring from different groups. (I) The hydroxyproline content of the liver in the offspring from different groups. *n* = 10. Data are presented as median with range (F and G) or mean ± SEM (A–E, H, I), **p < 0.01, ***p < 0.001.

Offspring from control and SD mothers were fed with either a normal diet or an HFHC diet for 16 weeks (Figure 1A). We first evaluated the phenotypes of the male offspring. The liver weight was significantly increased in SD offspring compared to control offspring when fed with an HFHC diet (Figure 1B). A similar pattern was observed for TG measurements among the four groups (Figure 1C). In addition, the serum levels of ALT and AST in SD offspring were significantly higher than those in control offspring after HFHC feeding (Figure 1D). Histological analysis revealed that though all the HFHC‐fed offspring showed obvious hepatic lipid accumulation and fibrosis, SD offspring presented significantly exacerbated phenotypes compared to control offspring (Figure 1E–H). Quantification of the liver hydroxyproline (a fibrosis marker) further supported exacerbated hepatic fibrosis in HFHC‐fed offspring born from SD mothers (Figure 1I). Considering the well‐documented association between early metabolic dysfunction and subsequent NASH development [29, 30], we further characterised early‐life metabolic parameters in 4‐week‐old offspring. The results indicated no significant differences in glucose tolerance, body fat composition, gas exchange, energy consumption, daily food and water intake between the two groups (Figure S1A–I). Moreover, we performed GTT in control and sleep‐deprived pregnant mice at E17.5 and revealed no significant difference in glucose tolerance between the two groups (Figure S1J). These results indicate that the increased susceptibility to NASH of SD offspring is not associated with early‐life metabolic disturbances in offspring.

To further examine whether the impact of maternal SD on offspring health differs by sex, we further compared the phenotypes in female offspring mice fed with an HFHC diet. Compared to control female offspring, exacerbated NASH phenotypes were observed in the female offspring from SD mothers, as evidenced by higher liver weight (Figure S2A), excessive hepatic TG accumulation (Figure S2B), and increased serum levels of ALT and AST (Figure S2C). Histological evaluations revealed more pronounced fat deposition and fibrosis in the SD offspring compared to control offspring (Figure S2D), with higher scores of steatosis and inflammation, and a greater extent of fibrosis (Figure S2E–G). Together, maternal SD predisposes their male and female offspring to diet‐induced NASH development in adulthood.

### 3.2 Maternal Sleep Deprivation During Pregnancy Increased Hepatic Inflammation and Apoptosis in HFHC‐Fed Offspring

**FIGURE 2:**
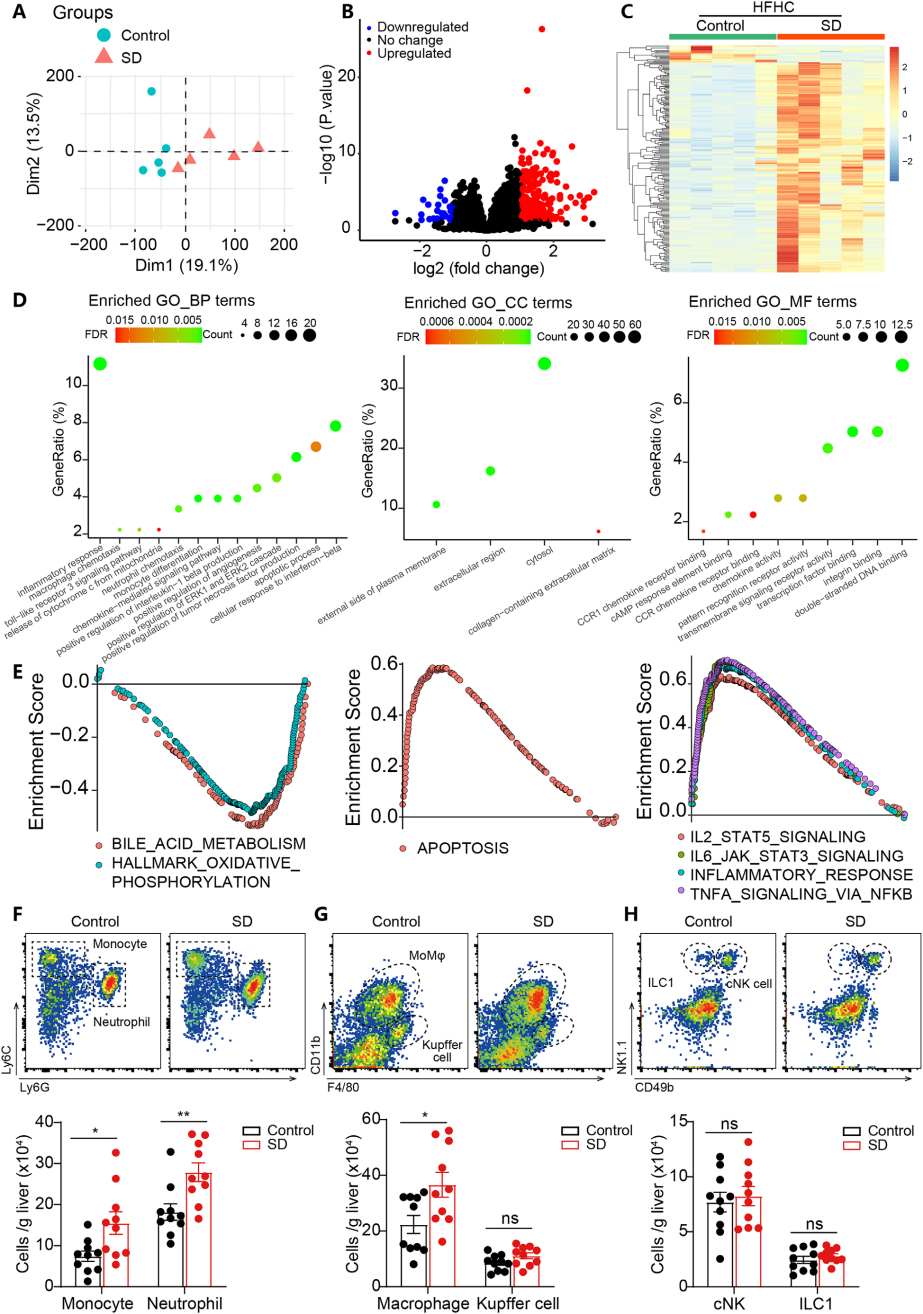
Maternal sleep deprivation during pregnancy increased hepatic inflammation and apoptosis in HFHC‐fed male offspring. (A) RNA sequencing was performed to compare gene expression in the male offspring liver tissues from two groups. Principal component analysis (PCA) suggested a clear separation between control and SD offspring. *n* = 5. (B) Volcano plot of the RNA‐seq results showing differentially expressed genes (DEGs). The red dots represent significantly upregulated genes, and the blue dots represent significantly downregulated genes. (C) Heatmap plot showing the relative expression of DEGs in liver tissues of offspring from two groups. (D) GO enrichment analysis results. (E) Gene set enrichment analysis results using the entire RNA‐seq dataset. (F‐H) Flow cytometry images and quantitative analysis of monocyte, neutrophil, macrophage, Kupffer cell and NK cell numbers in the offspring liver tissues from two groups. *n* = 10. Data are mean ± SEM, *p < 0.05, **p < 0.01.

To determine the potential mechanisms and signalling pathways responsible for the augmented hepatic damage observed in the SD offspring, we performed RNA sequencing analysis on liver tissues from HFHC‐fed male offspring. Principal component analysis showed that the samples from SD offspring were clearly separated from control offspring (Figure 2A). We identified 201 DEGs between control and SD offspring (Figure 2B,C). Gene ontology (GO) analysis results indicated enriched terms related to inflammatory response, cell chemotaxis, chemokine activity, and apoptosis process in the SD group (Figure 2D). Gene set enrichment analysis (GSEA) results confirmed the increased inflammation and apoptosis pathways in the SD offspring (Figure 2E). Moreover, GSEA results indicated the downregulation of metabolism‐related pathways such as oxidative phosphorylation (Figure 2E), in agreement with increased TG accumulation and hepatic steatosis in SD offspring (Figure 1C,F).

To validate these RNA‐seq results, we performed flow cytometry analysis to quantify the number of monocytes, neutrophils, macrophages, Kupffer cells, and NK cells accumulated in liver tissues. The results revealed a significantly higher number of monocytes, neutrophils, and macrophages accumulated in the liver of SD offspring compared to those of control offspring (Figure 2F–H), accompanied by increased inflammatory/chemotactic cytokine levels (Figure S3A). TUNEL staining results suggested enhanced hepatocyte apoptosis in SD offspring compared to control offspring (Figure S3B). Consistently, SD offspring exhibited increased pro‐apoptotic proteins, including cleaved CASPASE3 and BAX, and reduced levels of anti‐apoptotic BCL‐2 expression in the liver (Figure S3C). In addition to these findings in male offspring, we further confirmed an enhanced inflammatory response and activation of apoptotic pathways in HFHC‐fed female offspring born to SD mothers compared with controls (Figure S4A,B), while the key components of the oxidative phosphorylation complexes, including Ndufs3, Uqcrfs1, Cox5a, and Cox5b, were markedly reduced (Figure S4C). Together, these consistent results in both male and female offspring of SD mothers indicate that maternal SD predisposes the offspring to NASH progression through enhanced inflammatory responses, increased apoptosis, and impaired oxidative phosphorylation.

### 3.3 Hepatocytes From SD Offspring Were More Susceptible to Palmitate Acid‐Induced Injury

**FIGURE 3:**
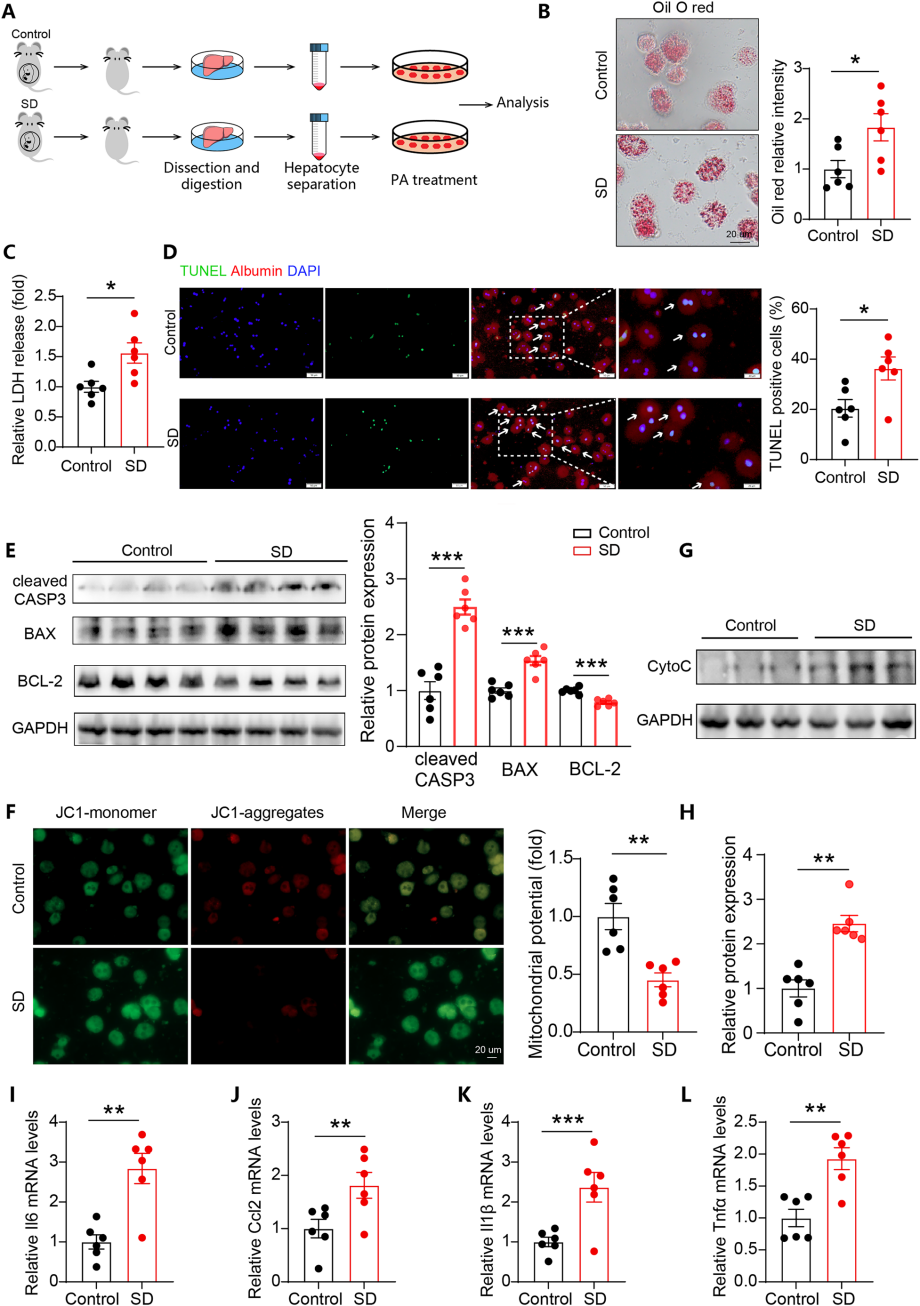
Maternal sleep deprivation exacerbated lipotoxic injury induced by palmitate acid in offspring hepatocytes. (A) Schematic illustration showing the study protocol. (B) Representative oil red O staining images and quantitative analysis between the indicated groups. Scale bar represents 20 um. (C) Relative lactate dehydrogenase (LDH) release in the offspring hepatocytes from two groups. (D) TUNEL staining results and quantitative analysis of hepatocellular apoptosis in the offspring from two groups. Scale bar represents 50 um. (E) WB results showing apoptotic‐related protein levels in liver tissues from the indicated groups. (F) JC‐1 staining results reflecting mitochondrial membrane potential in the offspring hepatocytes from the indicated groups. Scale bar represents 20 um. (G, H) WB results showing cytochrome C (Cyto C) levels in cytoplasm in the indicated groups. (I–L) Expression of inflammatory cytokines in the offspring hepatocytes from the indicated groups. *n* = 6. Data are mean ± SEM, *p < 0.05, **p < 0.01, ***p < 0.001.

To gain a more direct effect of gestational SD on offspring hepatocyte health, we isolated primary hepatocytes from offspring and treated them with palmitic acid (PA) (Figure 3A). Consistent with the animal experiment results, oil O staining revealed increased lipid accumulation in hepatocytes from SD offspring (Figure 3B). Compared to hepatocytes from control offspring, hepatocytes from SD offspring released significantly higher levels of LDH after PA treatment (Figure 3C). TUNEL staining suggested an increase in the hepatocyte apoptotic proportion in the SD group (Figure 3D). Consistently, WB analysis confirmed reduced BCL‐2 levels and increased levels of BAX and cleaved CASPASE3 in hepatocytes from SD offspring (Figure 3E). The decrease in mitochondrial membrane potential is also considered one of the early signs of cell apoptosis [31]. Compared with the control group, the mitochondrial membrane potential was considerably decreased in hepatocytes from SD offspring (Figure 3F). Meanwhile, there was a significant increase in cytochrome c release in the cytoplasm in SD offspring, supporting the instability of mitochondrial membrane potential (Figure 3G,H). Moreover, inflammatory markers including *Il6, Ccl2, Il‐1β* and *Tnfα* were significantly higher in hepatocytes from SD offspring compared to the control group (Figure 3I–L). Collectively, these data indicated that hepatocytes from SD offspring were more susceptible to lipotoxic injury.

### 3.4 NR4A3 Upregulation Contributed to the Increased Susceptibility to Lipotoxic Injury in Hepatocytes From SD Offspring

**FIGURE 4:**
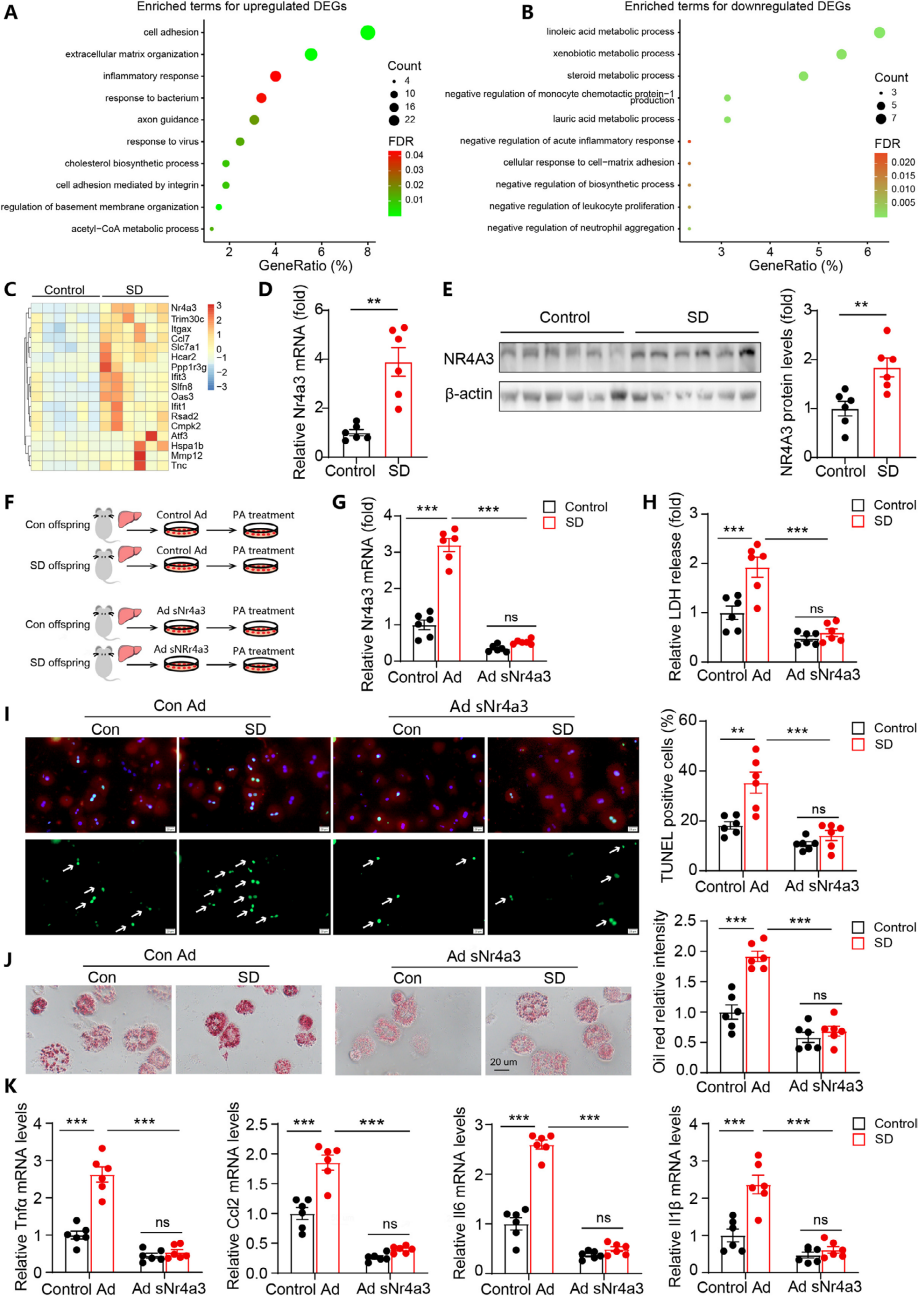
NR4A3 upregulation contributed to the increased susceptibility to lipotoxic injury in hepatocytes from SD offspring. (A, B) GO enrichment analysis of differentially expressed genes (DEGs) in livers at baseline between control and SD offspring groups. *n* = 6. (C) We intersected DEGs in the liver of offspring at baseline and those under HFHC feeding, and a total of 17 common DEGs were identified. The relative expression of these 17 DEGs in the two groups at baseline were shown as a heatmap. (D) Relative *Nr4a3* mRNA expression levels in liver tissues from the two groups. (E) WB results showing NR4A3 protein levels in liver tissues from the two groups. (F) Schematic illustration showing the primary hepatocytes experimental protocol. (G) Relative *Nr4a3* mRNA expression levels in primary hepatocyte in the indicated groups. (H) Comparison of LDH release in the indicated groups. (I) TUNEL staining results and quantitative analysis of cellular apoptosis. White arrows indicate apoptotic nucleus. Scale bar represents 20 um. (J) Representative oil red O staining images and quantitative analysis among the indicated groups. Scale bar represents 20 um. (K) Expression of inflammatory cytokines in the offspring hepatocytes from the indicated groups. *n* = 6. Data are mean ± SEM, **p < 0.01, ***p < 0.001.

To explore the mechanisms underlying the observed phenotype in SD offspring, we further performed RNA‐seq analysis using the offspring liver before initiating the HFHC diet. Functional enrichment analysis of the DEGs indicated upregulated terms including cell adhesion, inflammatory response, and cholesterol biosynthetic process in SD offspring at baseline (Figure 4A), while downregulated DEGs were enriched in terms related to metabolic process (Figure 4B). To further identify key molecular mediators, we intersected DEGs at baseline with DEGs under HFHC feeding and revealed 17 common DEGs (Figure 4C). Among them, a transcription factor nuclear receptor subfamily 4 group A member 3 (NR4A3) was highly upregulated in liver tissues from SD offspring, and its upregulation was further confirmed by WB and PCR analysis in both male (Figure 4D,E) and female offspring (Figure S4D,E). NR4A3 plays an important role in promoting inflammation [32, 33], oxidative stress [34], and cellular apoptosis [35]. Moreover, NR4A3 plays a key role in promoting the pathogenesis of NASH by activating inflammatory pathways and increasing hepatic steatosis [36]. Therefore, we tested whether NR4A3 upregulation mediated the exacerbated hepatocyte injury in SD offspring.

To test this hypothesis, primary hepatocytes from SD and control offspring were treated with PA in the presence of control adenovirus or NR4A3‐silencing adenovirus (Figure 4F,G). In the control adenovirus setting, PA treatment induced significantly greater LDH release in hepatocytes from SD offspring than from controls (Figure 4H). However, when NR4A3 was silenced, no significant difference in LDH release between the two groups was observed (Figure 4H). Moreover, the augmented apoptosis, lipid deposition, and expression of proinflammatory genes in PA‐treated hepatocytes from SD offspring were diminished in the setting of NR4A3 deficiency (Figure 4I–K). These results suggest that the increased susceptibility to lipotoxic injury in SD offspring hepatocytes was dependent on increased NR4A3 expression.

### 3.5 Maternal Melatonin Supplementation During Sleep Deprivation Alleviated the Increased Steatohepatitis Susceptibility in Offspring

**FIGURE 5:**
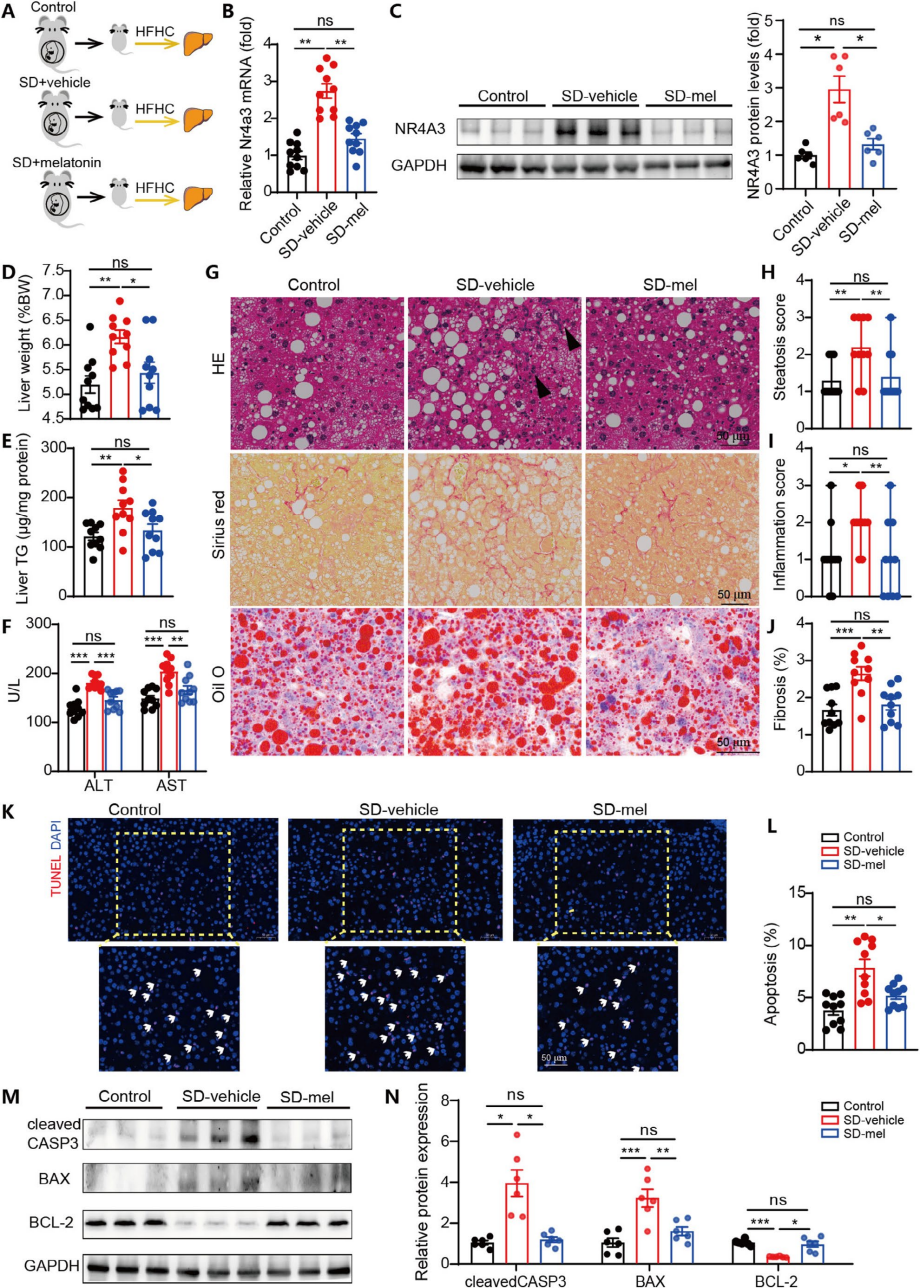
Maternal melatonin supplementation during sleep deprivation alleviated the increased steatohepatitis susceptibility in offspring. (A) Schematic illustration showing the experimental protocol. (B) Levels of *Nr4a3* mRNA expression levels in liver tissues from the indicated groups. *n* = 10. (C) WB results showing NR4A3 levels in liver tissues from the three groups. *n* = 10. (D–F) Comparison of liver weight/body weight ratios, liver TG contents, and serum levels of ALT and AST among the indicated groups. *n* = 10. (G) Representative images of the liver sections stained with HE, Sirius red and oil red O in the indicated groups. Scale bar represents 50 um. Black arrow heads indicate infiltrated immune cells. (H–J) Steatosis scores, inflammation scores, and fibrotic areas were calculated based on liver histological staining in the indicated groups. *n* = 10. (K, L) TUNEL staining results and quantitative analysis of hepatic apoptosis. *n* = 10. White arrows indicate apoptotic cells. Scale bar represents 50 um. (M, N) WB results showing apoptotic‐related protein levels in liver tissues from the three groups. *n* = 6. Data are mean ± SEM, *p < 0.05, **p < 0.01, ***p < 0.001.

Melatonin is a hormone secreted by the pineal gland, which plays key roles in regulating the biological clock and possess potent anti‐oxidative and anti‐inflammatory properties [20, 21]. Previous studies have revealed that maternal SD significantly affected the placental melatonin signalling [23]. Therefore, we sought to explore whether melatonin administration during SD pregnancy could alleviate the increased steatohepatitis susceptibility in offspring (Figure 5A). Compared to vehicle treatment, maternal melatonin supplementation during SD significantly reversed the upregulated hepatic NR4A3 levels in offspring (Figure 5B,C). Meanwhile, maternal melatonin administration significantly alleviated the increase of liver weight, hepatic TG concentration, and levels of serum ALT and AST primed by maternal SD (Figure 5D–F). Moreover, maternal melatonin treatment significantly alleviated hepatic fat deposition, fibrosis, and inflammation scores (Figure 5G–J), as well as suppressed hepatic apoptosis in SD offspring (Figure 5K–N).

Consistent with these functional and histological analysis results, unbiased RNA‐seq analysis on liver tissues supported the protective effects of gestational melatonin supplementation. Melatonin treatment induced significant changes in hepatic gene expression in SD offspring with significant suppression of *Nr4a3* (Figure S5A,B). Enrichment analysis of DEGs indicated that downregulated genes by melatonin treatment were primarily involved in inflammatory response and cell chemotaxis, while upregulated genes were enriched in metabolism process (Figure S5C,D). In addition, GSEA using the entire transcriptomic dataset also confirmed the upregulation of lipid catabolism (e.g., fatty acid metabolism and oxidative phosphorylation) and the suppression of inflammation (Figure S5E,F). Consistent with the enrichment analysis results (e.g., suppressed neutrophil chemotaxis, macrophage chemotaxis, leukocyte chemotaxis) (Figure S5C), gestational melatonin administration significantly reversed the infiltration of monocytes, neutrophils, and macrophages in the livers of SD offspring (Figure S5G–J). These results suggested that the increased steatohepatitis susceptibility in SD offspring was alleviated by maternal melatonin treatment.

### 3.6 Maternal Melatonin Supplementation Improved Tolerance to Lipotoxic Injury in Offspring Hepatocytes

**FIGURE 6:**
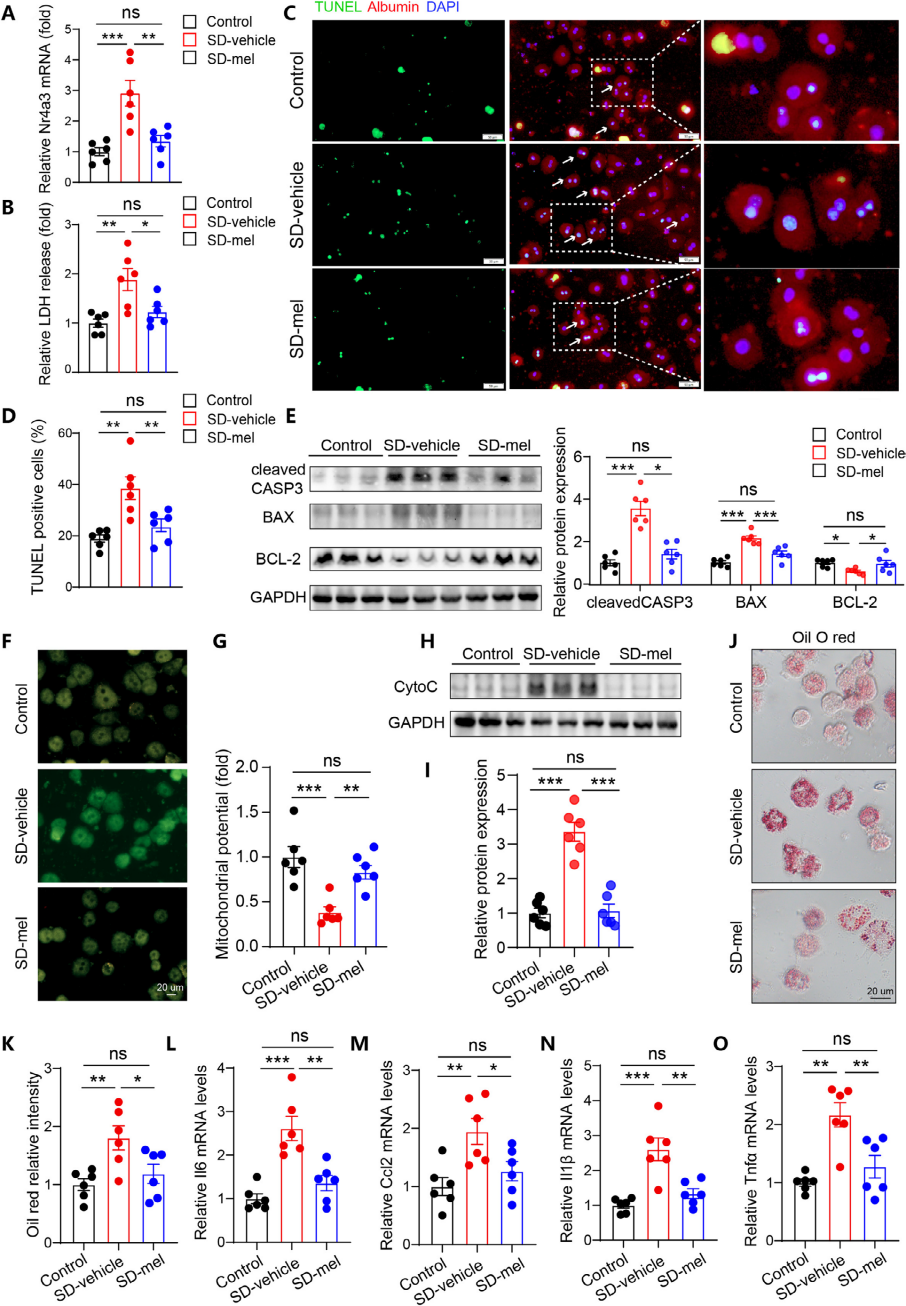
Maternal melatonin supplementation during sleep deprivation improved tolerance to lipotoxic injury of offspring hepatocytes. (A) Relative *Nr4a3* mRNA expression levels in primary hepatocytes among the three groups. (B) Relative LDH release in the offspring hepatocytes among the three groups. (C, D) TUNEL staining results and quantitative analysis of hepatocellular apoptosis in the offspring. Scale bar represents 50 um. (E) WB results showing apoptotic‐related protein levels in offspring hepatocytes. (F, G) JC‐1 staining results showing mitochondrial membrane potential in the offspring hepatocytes from the three groups. Scale bar represents 20 um. (H, I) WB results showing cytochrome C (Cyto C) protein levels in cytoplasm from the three groups. (J, K) Representative oil red O staining images and quantitative analysis among the indicated groups. Scale bar represents 20 um. (L–O) Expression of inflammatory genes in the offspring hepatocytes from the three groups. *n* = 6. Data are mean ± SEM, *p < 0.05, **p < 0.01, ***p < 0.001.

We further investigated whether maternal melatonin treatment during SD directly improved health of offspring hepatocytes. Compared to vehicle treatment, maternal melatonin treatment significantly decreased expression of *Nr4a3* in hepatocytes from SD offspring (Figure 6A). In addition, melatonin supplementation reduced PA‐induced LDH release (Figure 6B), and improved apoptosis in SD offspring hepatocytes (Figure 6C–E). Consistently, maternal melatonin supplementation preserved mitochondrial membrane potential and reduced cytochrome c release in comparison to vehicle treatment (Figure 6F–I). Additionally, melatonin administration during pregnancy significantly reduced lipid accumulation (Figure 6J,K), and suppressed inflammatory cytokine expression in SD offspring hepatocytes (Figure 6L–O).

Since maternal melatonin treatment reversed the upregulation of NR4A3 in SD offspring livers and hepatocytes, which played as a key mediator responsible for the enhanced lipotoxic injury in hepatocytes from SD offspring (Figure 4), we speculated that NR4A3 might be a potential mediator of melatonin‐induced protective effects. To test this hypothesis, we overexpressed NR4A3 in primary hepatocytes from both SD offspring and offspring born to SD mother treated with melatonin (SD‐Mel), to abolish the suppression of NR4A3 induced by gestational melatonin treatment (Figure S6A–C). Compared with control adenovirus (Adcon) infection in SD offspring hepatocytes, NR4A3 was significantly increased in hepatocytes from both SD and SD‐Mel offspring after infection with adenovirus encoding *Nr4a3* (AdNr4a3) (Figure S6C). Compared with Adcon‐infected SD offspring hepatocytes, NR4A3 overexpression in hepatocytes from both SD and SD‐Mel offspring led to increased lipotoxic injury (Figure S6D–G). However, no significant differences in lipotoxic injury were observed between AdNr4a3‐infected SD group and AdNr4a3‐infected SD‐Mel group (Figure S6D–G), suggesting that melatonin exerted its protective effects mainly through normalising NR4A3 expression in offspring hepatocytes.

## 4 Discussion

In the present study, we provided evidence that fetal exposure to maternal SD was associated with increased susceptibility to NASH in adulthood. First, histological experiments revealed that maternal SD during pregnancy promoted hepatic steatosis, fibrotic response, and inflammation in offspring in adulthood. In vitro studies further suggested that primary hepatocytes from SD offspring were more susceptible to lipotoxic injury. Second, unbiased RNA‐sequencing analysis indicated that maternal SD led to augmented activation of inflammatory and apoptosis pathways and suppressed energy metabolism pathways in offspring liver tissues, which were attributed to the upregulation of the transcription factor NR4A3. Third, maternal melatonin supplementation during pregnancy significantly alleviated the adverse effects of maternal SD on offspring liver health through inhibiting NR4A3. Collectively, these results suggest that maternal SD during pregnancy increased the risks of NASH development in offspring, and maternal melatonin supplementation may hold potential therapeutic promise.

The prevalence of NASH is increasing rapidly, and effective therapies managing NASH are still largely lacking. Most studies have focused on elucidating the pathogenic mechanisms of NASH in adulthood, but growing evidence indicates that its risk may originate from early‐life adverse exposures [37, 38, 39, 40, 41]. Human studies using magnetic resonance spectroscopy indicated that babies born to mothers with gestational diabetes showed a significant increase in liver fat [42]. Moreover, there are abundant animal models (rodent and nonhuman primates) to support the association between maternal obesity/obesogenic diet and offspring susceptibility to NASH [10, 43, 44, 45]. Unhealthy dietary habits, including maternal high fat diet [46, 47, 48], protein malnutrition [49], and low calcium intake [50] during pregnancy and lactation, induce peroxisome deficiency and dysregulated fatty acid metabolism in the offspring. Moreover, intrauterine exposure to synthetic glucocorticoids harmed TG metabolic pathways in the fetal liver [51]. Here, we extended our understanding of the developmental origins of NASH and provided the first evidence that the risk of NASH development in adult offspring is increased by maternal SD during pregnancy.

NASH is a complex multifactorial disease [52]. Steatosis is an early event in NASH development, and additional stresses such as lipotoxicity, oxidative stress, and inflammation synergistically induce hepatocyte death and fibrosis development [53, 54]. Chronic liver inflammation is considered the driving force to promote simple steatosis transition into NASH and beyond [55]. Through low‐grade yet persistent production of proinflammatory cytokines, including TNF, IL‐6, and IL‐1β, chronic liver inflammation can perpetuate hepatic healing processes, resulting in fibrosis that compromises immunosurveillance in the liver, thereby causing NASH progression and ultimately hepatic failure [56, 57, 58]. In our study, transcriptomic profiling and histological analysis revealed more serious pathological phenotypes of NASH in the offspring born to maternal SD. Notably, maternal SD was significantly associated with an intensified inflammatory response in the offspring liver, as functional enrichment analysis demonstrated that the most extensively upregulated pathways in SD offspring liver were related to immune response. Moreover, flow cytometry and qPCR analysis indicated enhanced myeloid cell infiltration and inflammatory gene expression in SD offspring liver. Consistently, other studies demonstrated that maternal SD also increased expression of inflammatory cytokines in the hippocampus [16, 59, 60], and elevated placental inflammation [61]. Together, these data suggest that maternal SD may increase NASH risks in offspring via a transcriptomic programming favouring inflammatory response and cellular injury.

Transcription factors regulate gene expression through specific binding to DNA sequences in gene promoters/enhancers via specialised domains [62]. Through RNA‐seq analysis, we identified NR4A3 as a significantly upregulated transcription factor in the SD offspring liver tissues. NR4A3 belongs to the NR4A family of nuclear orphan receptors [63, 64], which play important roles in energy metabolism [65], cardiovascular homeostasis [66] and immune response [67]. NR4A3 was reported to augment the inflammatory response in different disorders. In the development of arterial aneurysm, NR4A3 overexpression amplified the production of proinflammatory cytokines, chemokines, and reactive oxygen species, leading to enhanced vascular inflammation and aneurysm severity [68]. Similarly, deletion of NR4A3 in haematopoietic stem cells reduces inflammation in response to LPS‐induced stress, suggesting a pro‐inflammatory role of endogenous NR4A3 [69]. Of note, previous studies have reported that NR4A3 promotes the progression of NASH by increasing pro‐inflammatory genes and cellular apoptosis [36]. In the current study, our further analysis revealed that NR4A3 is an essential player in mediating the increased vulnerability to steatotic injury of SD offspring hepatocytes. Aligned with current findings, previous studies highlighted NR4A family as a set of genes associated with response to prenatal stress. For example, imposed food restriction during gestation is capable of exerting an adverse impact on later brain development via increasing the expression of NR4A1 and NR4A3 in the striatum of adolescent offspring [70]. Together, these data suggest that NR4A3 may represent a potential hub gene mediating the adverse effects of diverse intrauterine exposures on the long‐term health of offspring, which warrants more detailed investigations in the future.

Considering the prevalence of gestational SD and that NASH represents a common chronic liver disease, it might be imperative to take measures to reduce susceptibility to NASH in the offspring. Melatonin is a circadian hormone with antioxidant and anti‐inflammatory properties secreted by the pineal gland [71]. Melatonin secretion is pivotally affected by sleep and sleep disturbance [22], and the melatonin signalling in the placenta is suppressed by SD during pregnancy [23]. Interestingly, gestational melatonin supplementation can regulate the expression of cytokines and antioxidant enzymes to improve programmed cardiovascular dysfunction in the offspring exposed to suboptimal intrauterine environments [72, 73]. Moreover, melatonin supplementation also alleviated the inflammatory response in the brains of offspring born to maternal SD, leading to improved learning and memory in offspring [24]. Interestingly, gestational melatonin administration in obese mothers improved liver architecture and hepatic metabolism in the offspring [74, 75]. Although previous studies have similarly investigated maternal melatonin supplementation and its protective effects on offspring health, these were conducted in different maternal settings. Here, we observed that maternal melatonin treatment significantly alleviated NASH pathogenesis in the offspring of maternal SD by inhibiting NR4A3 expression and limiting hepatic inflammation, steatosis, and fibrosis during NASH development in the offspring. Since melatonin did not exert toxic effects on embryo development [76], it may be a potentially safe therapeutic candidate for SD mothers during pregnancy to improve their offspring's liver health and beyond.

## 5 Limitation

Our study has several limitations. First, the current study primarily focuses on the impact of SD in the third trimester of pregnancy on offspring liver health, and further investigation is required to examine the impact of maternal SD at different pregnancy stages. Second, the animal models used fail to fully mimic the complex pathogenesis of maternal SD in humans; thus, future studies with prospective or retrospective cohorts are warranted to confirm the association between maternal SD and risks of NASH in offspring. Third, it remains unclear whether melatonin acts primarily by directly modulating maternal physiology or via crossing the placental barrier to act on the fetus. Given its lipophilic properties, melatonin readily crosses the placenta and raises both maternal and fetal levels, complicating the separation of their effects. Moreover, placental melatonin receptors (MT1/MT2) are downregulated under maternal SD [23], and melatonin has been shown to enhance trophoblast survival and is negatively associated with pre‐eclampsia [71]. Thus, maternal supplementation may also improve offspring health indirectly through the restoration of placental function. Further studies, for example, using placenta‐specific melatonin receptor knockout models, will be needed to dissect the relative contributions of these compartments. Fourth, though our data obtained from mice suggested promising benefits of melatonin treatment during gestation, it remains an open question whether these findings are translatable to human beings.

## 6 Conclusion

Collectively, our findings demonstrate that maternal SD during pregnancy elevates the risk of NASH development in adult offspring through upregulating transcription factor NR4A3. Importantly, we provide preclinical evidence that maternal melatonin supplementation effectively mitigates the adverse hepatic consequences of maternal SD through inhibiting NR4A3. Furthermore, this study provides novel evidence underscoring the long‐term impact of maternal sleep status on offspring metabolic health.

## Author Contributions

Ling Gao initiated the study and developed the concept of the article. Fei Guo designed and performed experiments, analysed the data and wrote the manuscript. Zexin Yang analysed and interpreted the data. Junsen She analysed RNA‐seq data. Chen Fang and Yizhi Hu wrote and revised the article. Ling Gao and Hefeng Huang supervised the study.

## Conflicts of Interest

The authors declare no conflicts of interest.

## Supporting information


**Figure S1:** Maternal sleep deprivation minimally affected early‐life metabolic features in offspring.
**Figure S2:** Maternal sleep deprivation during pregnancy promoted the development of NASH in female offspring.
**Figure S3:**. Maternal SD during pregnancy increased hepatic inflammation and apoptosis in HFHC‐fed male offspring.
**Figure S4:**. Maternal SD suppressed pathways related to oxidative phosphorylation and increased inflammation and apoptosis in female offspring.
**Figure S5:**. Maternal melatonin supplementation during sleep deprivation alleviated hepatic inflammation in offspring.
**Figure S6:** Gestational melatonin supplementation alleviates lipotoxic injury in hepatocytes of SD offspring via an NR4A3 dependent manner.

## Data Availability

Data will be made available on request.
