# Supplementary material for: Gestational Melatonin Supplementation Attenuates Maternal Sleep Deprivation‐Induced Steatohepatitis Susceptibility in Offspring
Source: Cell Prolif. 2025 Oct 28;59(5):e70138. doi: 10.1111/cpr.70138 (PMC13114791; doi:10.1111/cpr.70138)
Supplement: Supplementary file 1 — Figure S1: Maternal sleep deprivation minimally affected early‐life metabolic features in offspring. Figure S2: Maternal sleep deprivation during pregnancy promoted the development of NASH in female offspring. Figure S3:. Maternal SD during pregnancy increased hepatic inflammation and apoptosis in HFHC‐fed male offspring. Figure S4:. Maternal SD suppressed pathways related to oxidative phosphorylation and increased inflammation and apoptosis in female offspring. Figure S5:. Maternal melatonin supplementation during sleep deprivation alleviated hepatic inflammation in offspring. Figure S6: Gestational melatonin supplementation alleviates lipotoxic injury in hepatocytes of SD offspring via an NR4A3 dependent manner. [file CPR-59-e70138-s001.docx]

**SUPPLEMENTAL MATERIAL**

**Gestational Melatonin Supplementation Attenuates Maternal Sleep Deprivation-Induced Steatohepatitis Susceptibility in Offspring**

Fei Guo^a,b,1^, Zexin Yang^a,b,1^, Junsen She^c,d^, Chen Fang^d^, Yizhi Hu^d^, Hefeng Huang^a,b,c,d,e*^, Ling Gao^a,b,e*^

^a^ Obstetrics and Gynecology Hospital, Institute of Reproduction and Development, Fudan University

^b^ Shanghai Key Lab of Reproduction and Development, Shanghai Key Lab of Female Reproductive Endocrine Related Diseases, 200433, Shanghai, China

^c^ International Institutes of Medicine, the Fourth Affiliated Hospital, Zhejiang University School of Medicine, Yiwu, China

^d^ Institute of Medical Genetics and Development, Key Laboratory of Reproductive Genetics (Ministry of Education) and Women's Hospital, Zhejiang University School of Medicine, Zhejiang, China

^e^ Research Units of Embryo Original Diseases, Chinese Academy of Medical Sciences (No. 2019RU056), Shanghai, China

*Correspondence to: Ling Gao (gaoling@fudan.edu.cn) or Hefeng Huang (huanghefg@fudan.edu.cn)

^1^ Fei Guo and Zexin Yang Contributed equally.

**Supporting Information**

| **File name** | **Description** |
| --- | --- |
|  | **Supplementary figure S1**. Maternal sleep deprivation minimally affected early-life metabolic features in offspring. |
|  | **Supplementary figure S2** Maternal sleep deprivation during pregnancy promoted the development of NASH in female offspring. |
| **Supplemental Material** | **Supplementary figure S3**. Maternal SD during pregnancy increased hepatic inflammation and apoptosis in HFHC-fed male offspring. |
|  | **Supplementary figure S4**. Maternal SD suppressed pathways related to oxidative phosphorylation and increased inflammation and apoptosis in female offspring. |
|  | **Supplementary figure S5**. Maternal melatonin supplementation during sleep deprivation alleviated hepatic inflammation in offspring. |
|  | **Supplementary figure S6.** Gestational melatonin supplementation alleviates lipotoxic injury in hepatocytes of SD offspring via an NR4A3 dependent manner. |

**Supplementary figure S1**


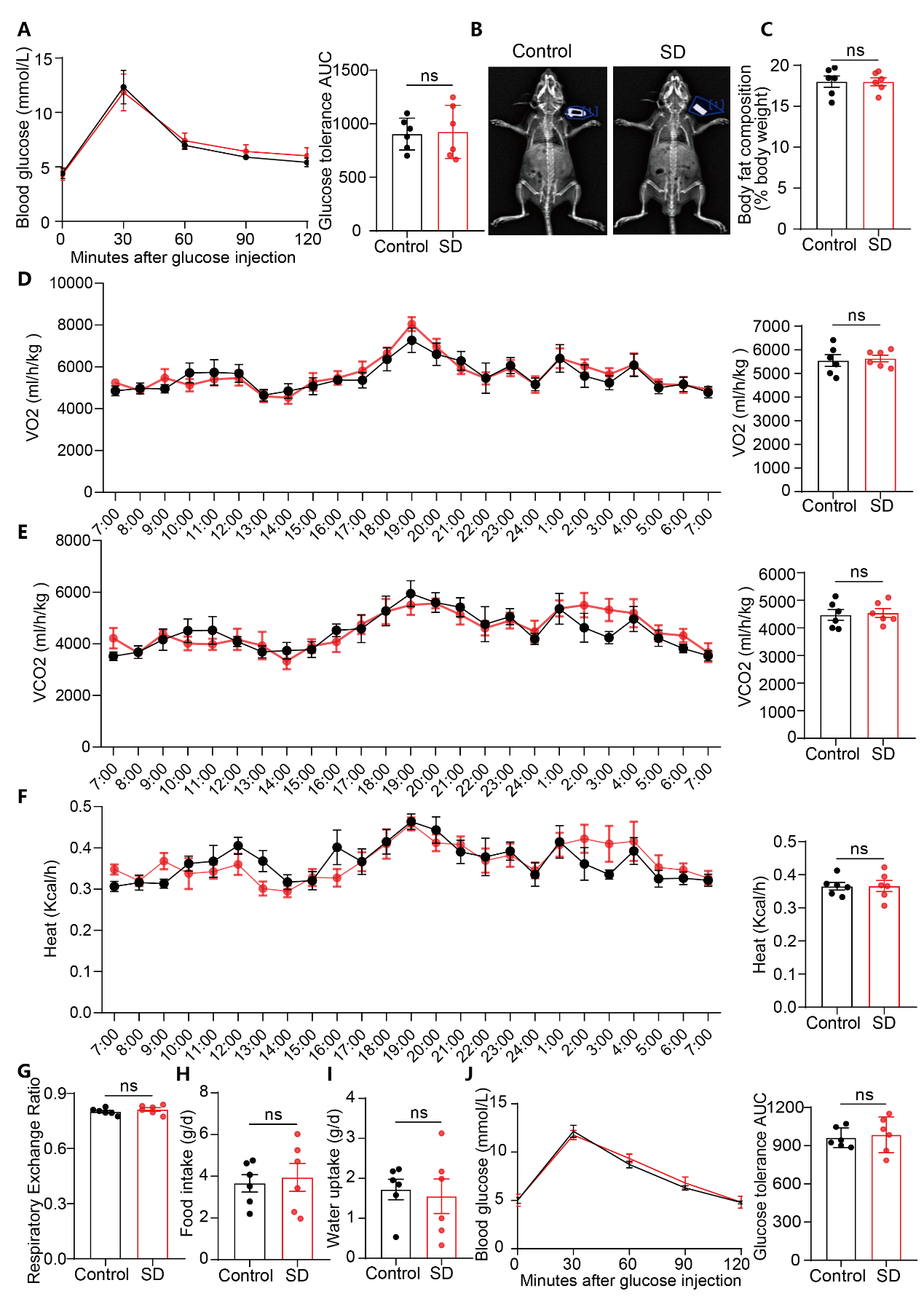


**Supplementary figure S1. Maternal sleep deprivation minimally affected early-life metabolic features in offspring.**

(A) Glucose tolerance test results in offspring from the two indicated groups. (B-C) Body fat composition measured by dual-energy X-ray absorptiometry in indicated groups. (D-I) Oxygen consumption rate (VO₂), carbon dioxide production rate (VCO₂), heat production, respiratory exchange ratio (RER), food intake, and water uptake in indicated groups. (J) Comparison of glucose tolerance test results in pregnant mice between the two groups. n = 6. Data are mean ± SEM, ns, no significant.

**Supplementary figure S2**
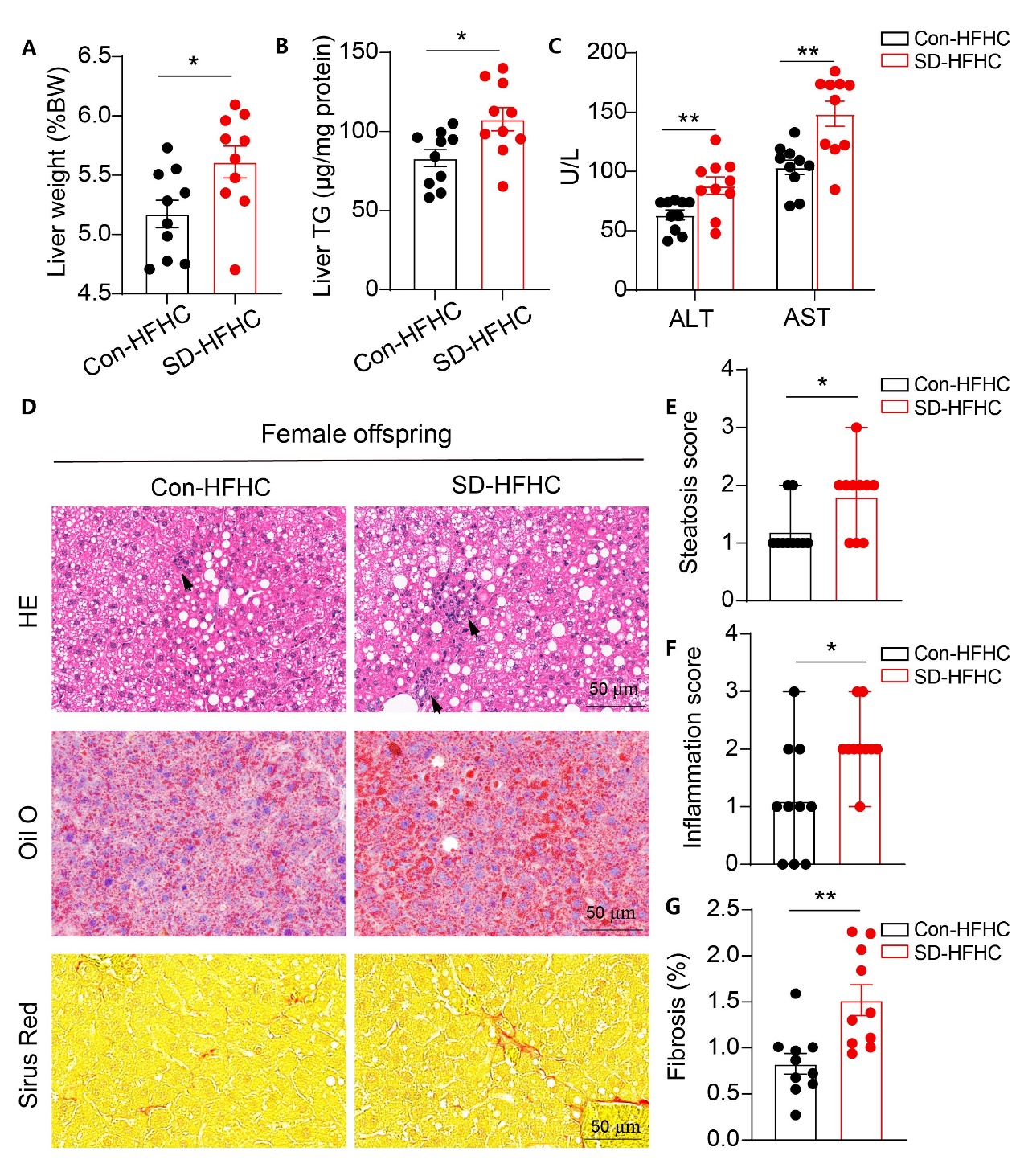


**Supplementary figure S2. Maternal sleep deprivation during pregnancy promoted the development of NASH in female offspring.**

(A-C) Comparison of liver weight/body weight ratios, liver TG contents, and serum levels of ALT and AST in the female offspring from the two groups. (D) Representative images of the liver sections stained with HE, oil red O, and Sirus red staining in the female offspring in indicated groups. Scale bar represents 50 um. Black arrow heads indicate infiltrated immune cells. (E-F) Steatosis scores and inflammation scores based on histological staining results in the indicated groups. (G) Comparison of fibrotic areas in the liver between the indicated groups. n = 10. Data are presented as median with range (E and F) or mean ± SEM (A-C, G), *p < 0.05, **p < 0.01.

**Supplementary figure S3**


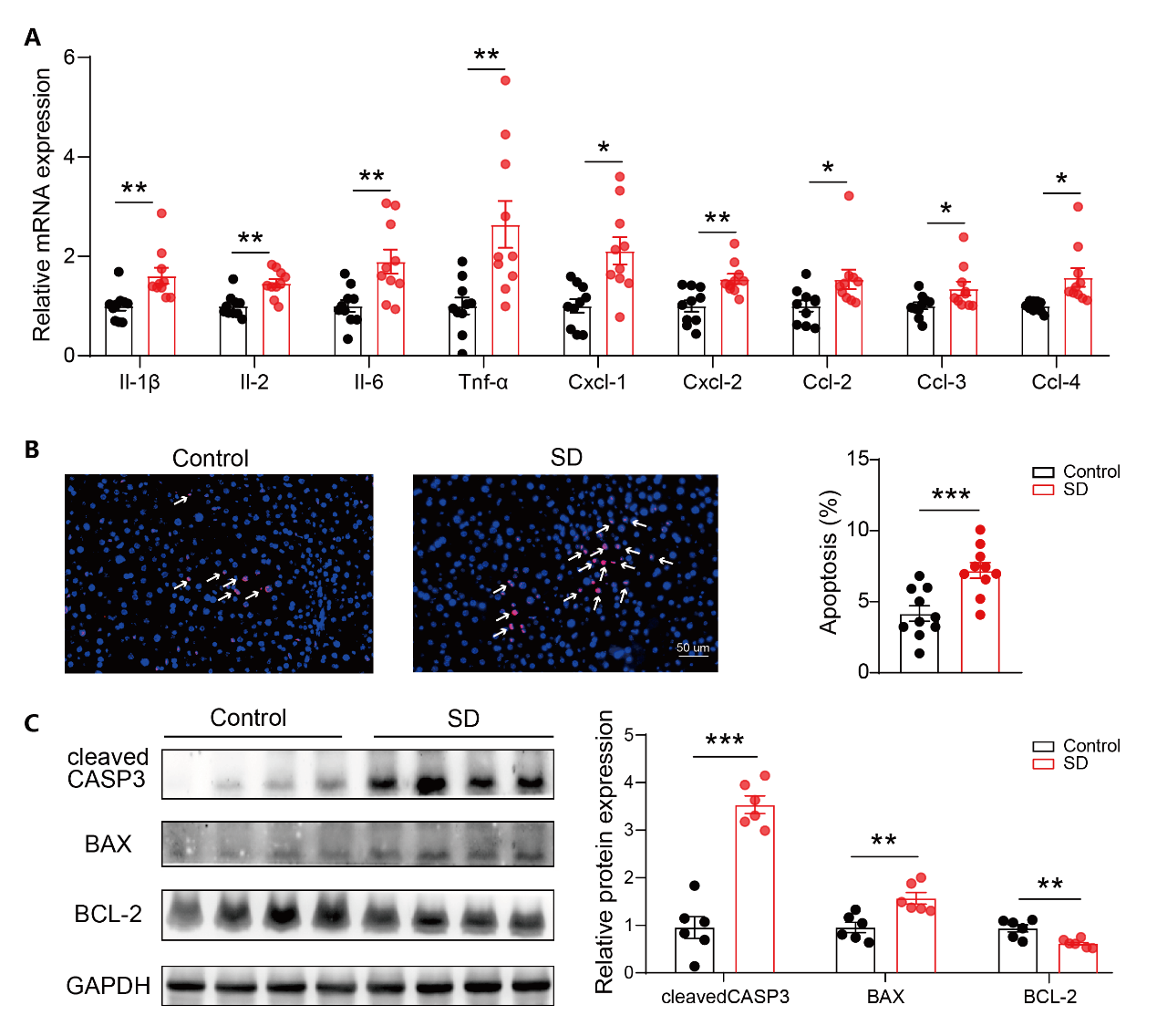


**Supplementary figure S3. Maternal SD during pregnancy increased hepatic inflammation and apoptosis in HFHC-fed male offspring.**

(A) Relative mRNA levels of inflammatory/chemotactic factors in the offspring liver tissues from the two groups. n =10. (B) TUNEL staining results and quantitative analysis of hepatic apoptosis in offspring from the two groups. n =10. Scale bar represents 50 um. (C) WB results showing apoptotic-related factor levels in liver tissues from the indicated groups n =6. Data are mean ± SEM, *p < 0.05, **p < 0.01, ***p < 0.001.

**Supplementary figure S4**


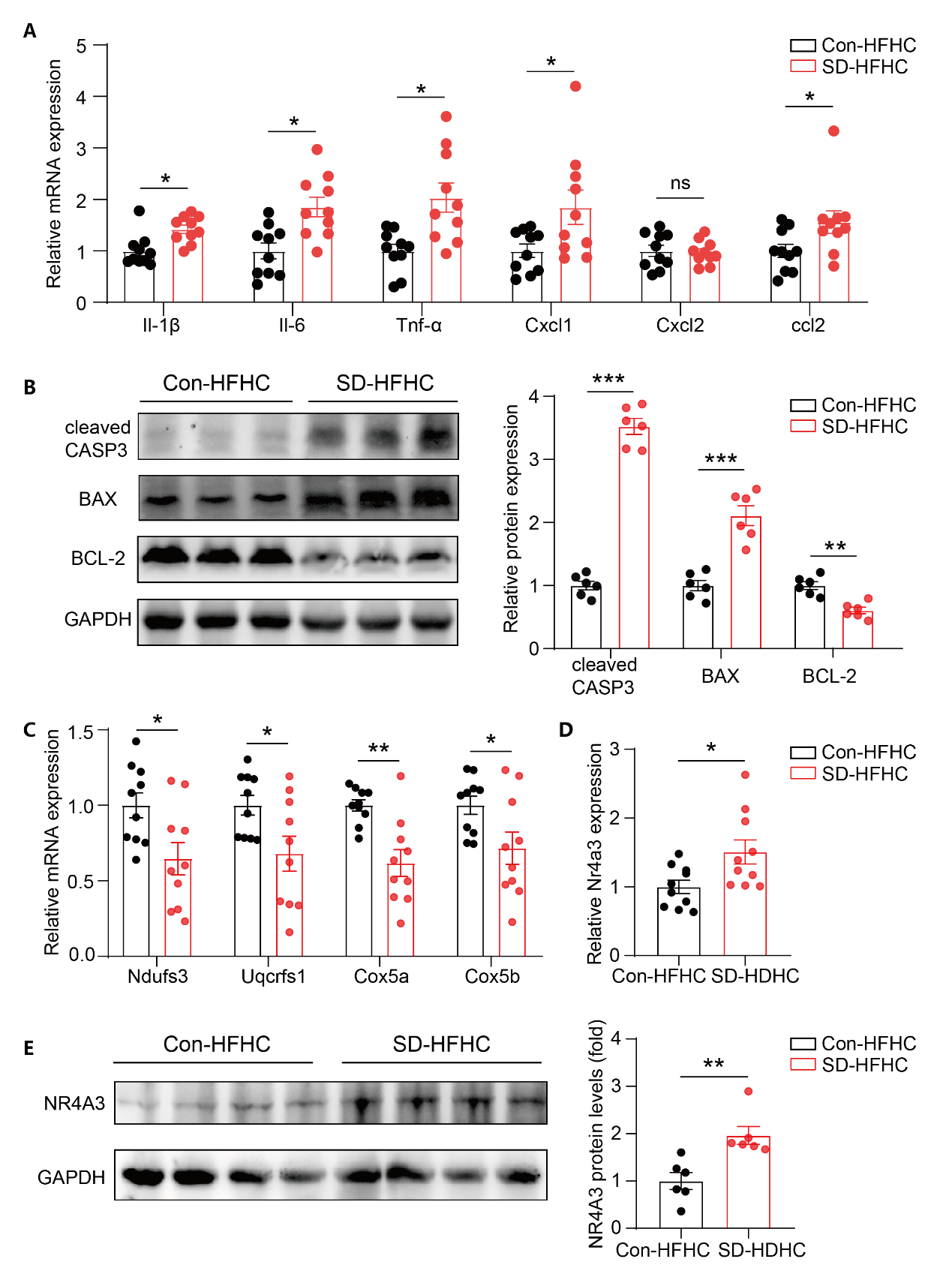


**Supplementary figure S4. Maternal SD suppressed pathways related to oxidative phosphorylation and increased inflammation and apoptosis in female offspring.**

(A) Relative mRNA levels of inflammatory/chemotactic-related factor in liver tissues from the two groups. n =10. (B) WB results showing apoptotic-related factor levels in liver tissues from the two groups. n =6. (C) Relative mRNA levels of mitochondrial complex components in liver tissues from the two groups. n =10. (D) Relative *Nr4a3* mRNA expression levels in liver tissues from the two groups. n =10. (E) WB results showing NR4A3 levels in liver tissues from the two groups. n = 6. Data are mean ± SEM, *p < 0.05, **p < 0.01, ***p < 0.001.

**Supplementary figure S5**


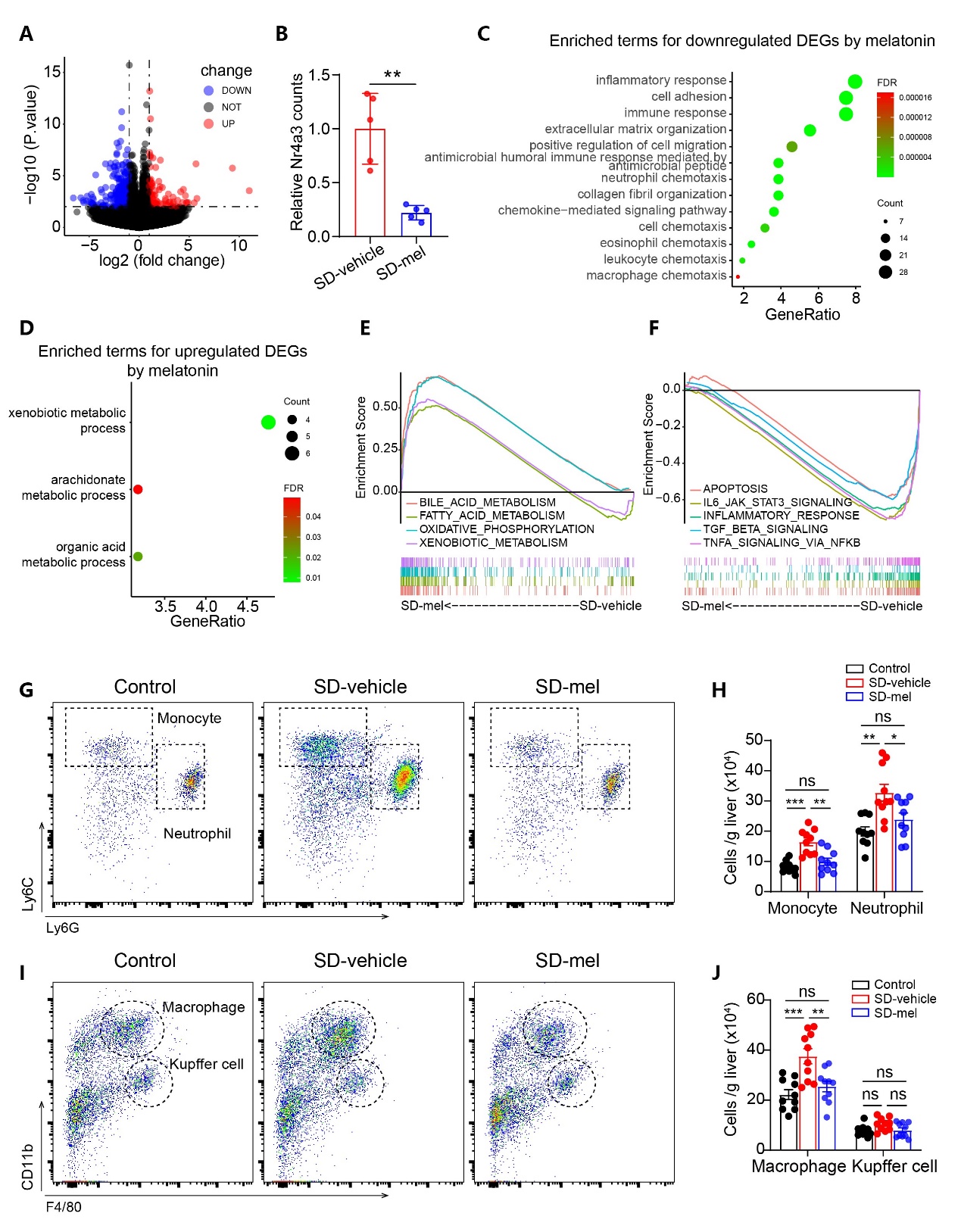


**Supplementary figure S5. Maternal melatonin supplementation during sleep deprivation alleviated hepatic inflammation in offspring.**

(A) RNA sequencing was performed to compare gene expression in the liver from SD-vehicle male offspring (born to SD mothers treated with vehicle) and SD-mel male offspring (born to SD mothers treated with melatonin). Volcano plot shows differentially expressed genes (DEGs) between the two groups. The red dots represent significantly upregulated genes, and the blue dots represent significantly downregulated genes. n=5. To avoid skewing of the plot, genes with an absolute value oflog2FoldChange>15 were omitted from the plot. (B) Comparison of Nr4a3 counts between the two groups based on the RNA-seq data. n=5. (C) GO enrichment analysis of DEGs downregulated by maternal melatonin treatment. (D) GO enrichment analysis of DEGs upregulated by maternal melatonin treatment. (E-F) Gene set enrichment analysis results using the entire RNA-seq dataset. (G-J) Flow cytometry images and quantitative analysis of monocyte, neutrophil, NK cell and Kupffer cell numbers in liver tissues of offspring from the indicated groups. n = 10. Data are mean ± SEM, *p < 0.05, **p < 0.01, ***p < 0.001.

**Supplementary figure S6**


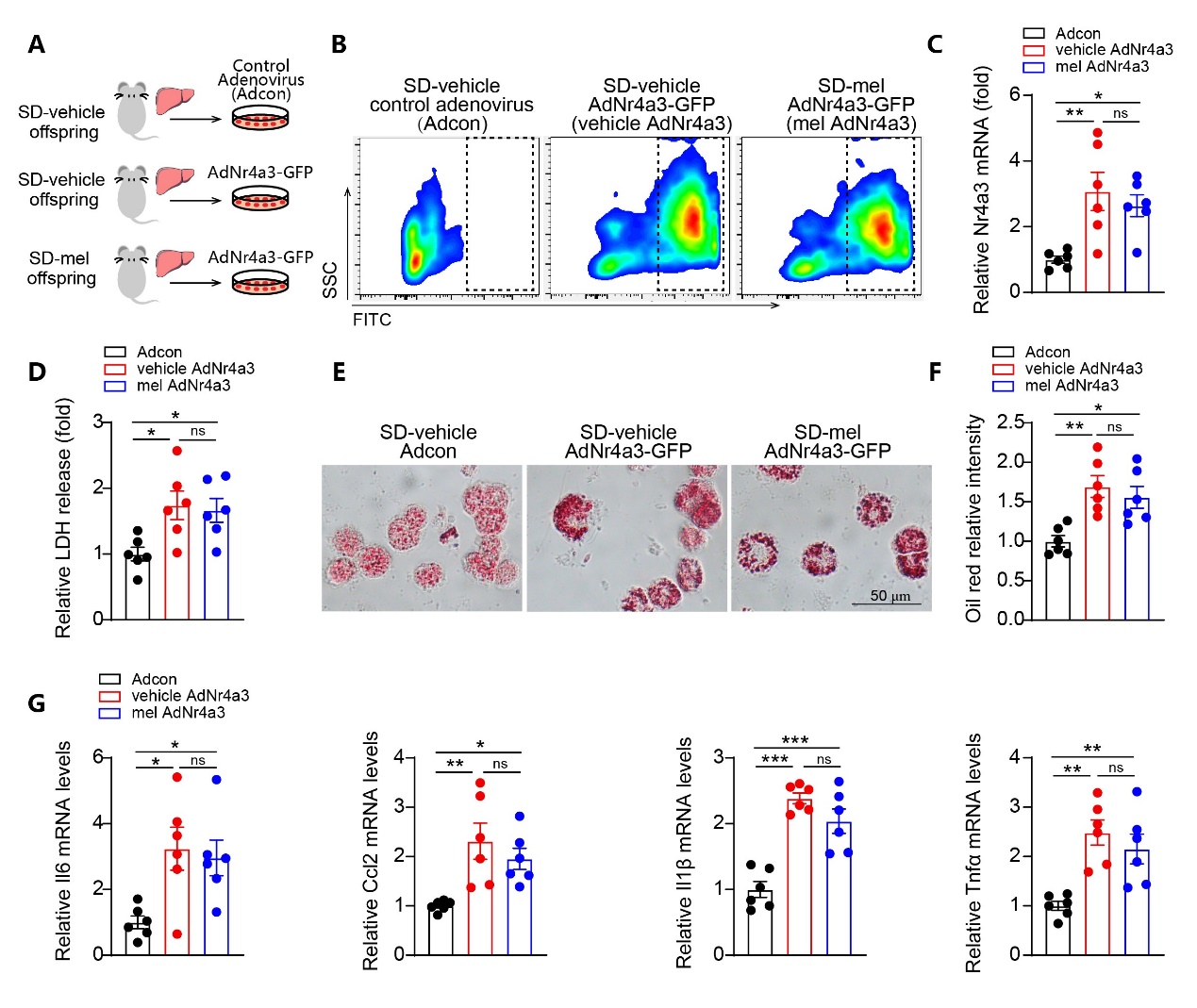


**Supplementary figure S6. Gestational melatonin supplementation alleviates lipotoxic injury in hepatocytes of SD offspring via an NR4A3 dependent manner.**

(A) Schematic illustration showing the allocation protocol. (B) Flow cytometry results showing successful infection of adenovirus encoding Nr4a3-GFP in hepatocytes. (C) Relative *Nr4a3* mRNA expression levels in primary hepatocyte in the indicated groups. (D) Comparison of LDH release in the indicated groups. (E-F) Representative Oil red O staining images and quantitative analysis among the indicated groups. Scale bar represents 50 um. (G) Expression of inflammatory cytokines in the hepatocytes from the indicated groups. n = 6. Data are mean ± SEM, *p < 0.05, **p < 0.01, ***p < 0.001.
